# Supplementary material for: Achromatic light patterning and improved image reconstruction for parallelized RESOLFT nanoscopy
Source: Sci Rep. 2017 Mar 20;7:44619. doi: 10.1038/srep44619 (PMC5357911; doi:10.1038/srep44619)
Supplement: Supplementary Information [file srep44619-s1.pdf]

Supplementary information for

Achromatic light patterning and improved image reconstruction for parallelized RESOLFT nanoscopy

Andriy Chmyrov[1,2,§†], Marcel Leutenegger[1†], Tim Grotjohann[1], Andreas Schönle[2], Jan Keller-Findeisen[1], Lars Kastrup[2], Stefan Jakobs[1,3], Gerald Donnert[2], Steffen J. Sahl[1] & Stefan W. Hell[1*]

[1] Max Planck Institute for Biophysical Chemistry, Department of NanoBiophotonics, Am Faßberg 11, 37077 Göttingen, Germany.
[2] Abberior Instruments GmbH, Hans-Adolf-Krebs-Weg 1, 37077 Göttingen, Germany.
[3] University of Göttingen, Medical Faculty, Department of Neurology, Robert-Koch-Str. 40, 37075 Göttingen, Germany.
[§] Present address: Helmholtz-Zentrum München, Institute of Biological and Medical Imaging, Ingolstädter Landstraße 1, 85764 Neuherberg, Germany.

[†] These authors contributed equally.

[*]Correspondence should be addressed to S.W.H. (hell@nanoscopy.de).

Supplementary Figures

L_1 Achromat f = 40 mm (Qioptiq G322209000)
L_2 Achromat f = 100 mm (Thorlabs AC254-100-A)
$L_{3,4}$ Achromat f = 125 mm (Thorlabs AC254-125-A)
L_5 Achromat f = 200 mm (Thorlabs AC254-200-A)
$L_{6,7,8}$ Achromat f = 160 mm (Qioptiq G322352000)

D_1 Dichroic 390/485/593 nm bandpass (Chroma ZT390/485/593tpc-UF5)
D_2 Dichroic 580 nm edge BrightLine (Semrock FF580-FDi01-25x36)
D_3 Dichroic 387 nm long pass (Chroma T387lp-UF1)
F_1 Bandpass filter 550/88 nm BrightLine (Semrock FF01-550/88-25)
F_2 Bandpass filter 641/75 nm BrightLine (Semrock FF02-641/75-25)

$G_{1,2}$ Binary phase grating, 36 µm period, 1:1 duty cycle (Laser-Laboratorium Göttingen)
PBS Polarizing beam splitter (B. Halle PTW 25)
λ/2 Achromatic half-wavelength retarder plate (Thorlabs AHWP05M-600)
OS Order selection mask, 4× Ø1 mm hole on Ø3 mm circle

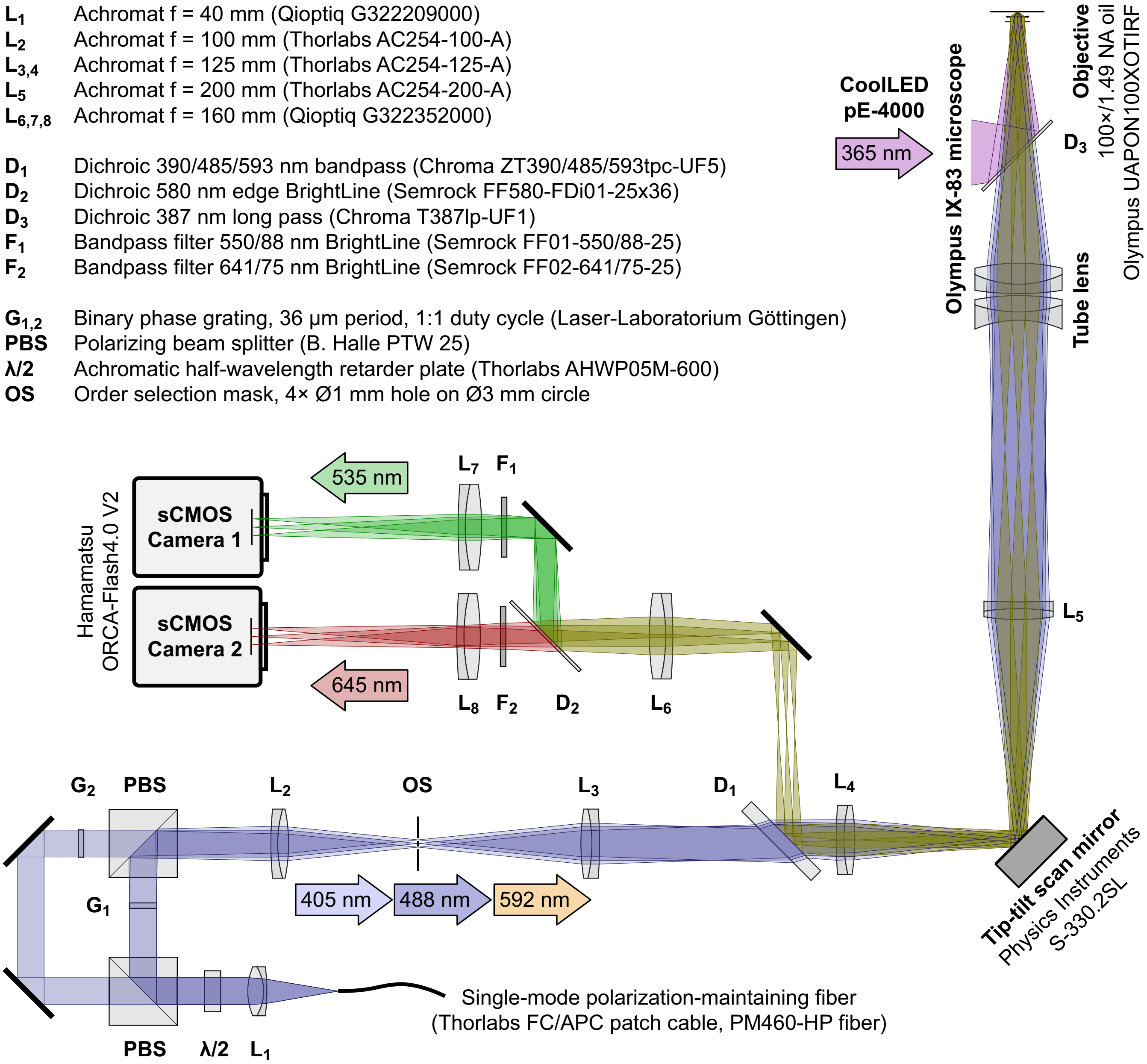

Supplementary Figure 1 | Scheme of the two-colour parallelized RESOLFT setup. The microscope features three patterned illuminations at 405, 488 and 592 nm wavelengths; a homogeneous illumination at 365 nm wavelength; and two fluorescence detection channels with 500–575 nm and 610–680 nm wavelength ranges.

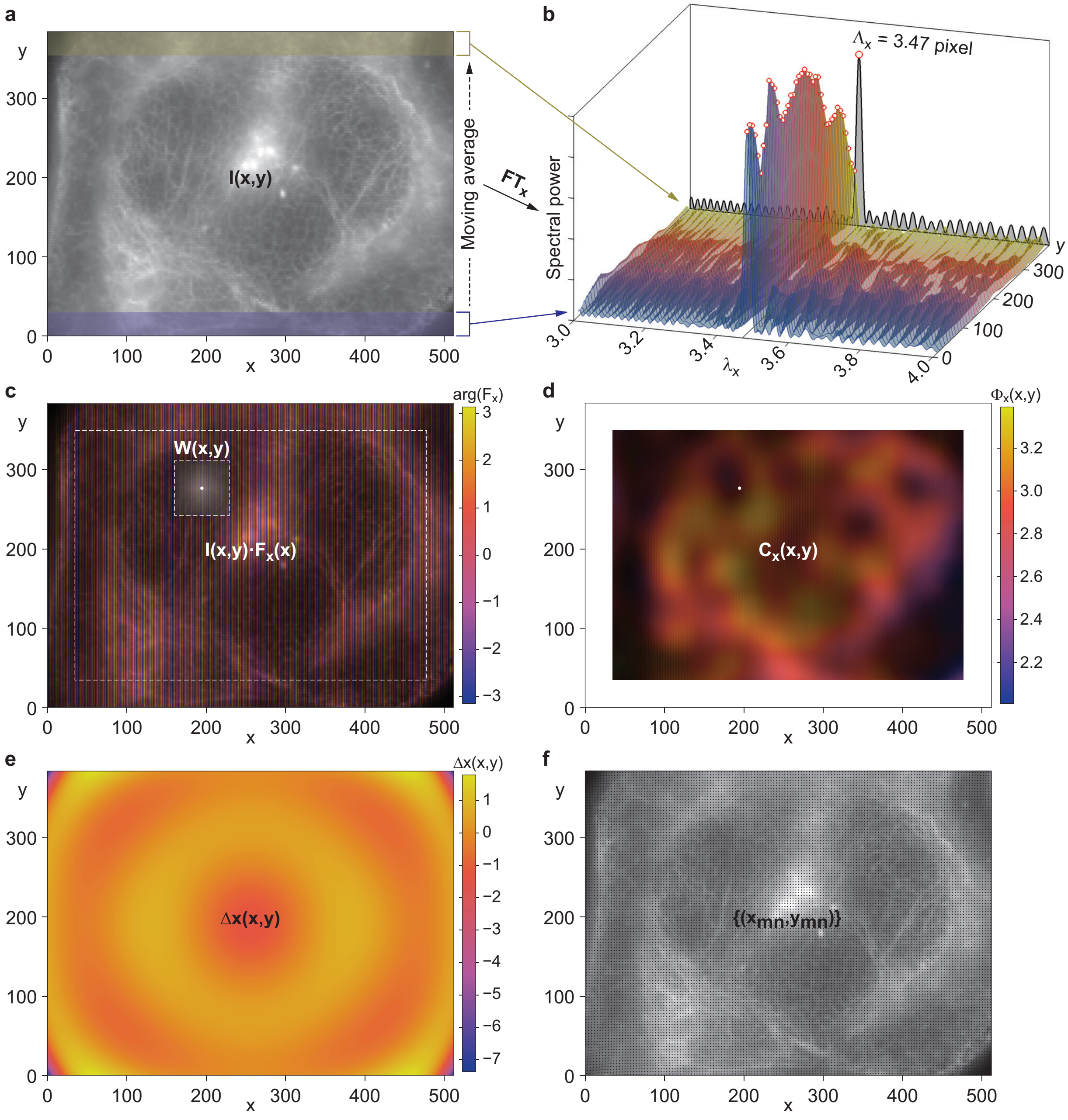

Supplementary Figure 2 | Localization of the signal origins. The illumination periods are estimated globally and the position offsets locally. (**a**) Sum image $I(x,y)$ and regions of the profiles $I_x(x,y)$ whose spectral powers (**b**) are analysed to find the horizontal period Λ_x. (**c**) Image $I(x,y)$ modulated by $F_x(x)$ and exemplary correlation window $W(x,y)$ with central point. The modulation phase is encoded as colour and the image intensity as brightness. (**d**) Local correlations $C_x(x,y)$. The unwrapped correlation phase $\Phi_x(x,y)$ is encoded as colour and the correlation amplitude as brightness. (**e**) Zernike-filtered and -extrapolated local position offset $\Delta x(x,y)$. (**f**) Image overlaid with the localized signal origins $\boldsymbol{X} = \{(x_{mn}, y_{mn})\}$ (black dots). Units: $x, y, \lambda_x, \Lambda_x$ and Δx in pixels; phases $\arg F_x$ and Φ_x in radians.

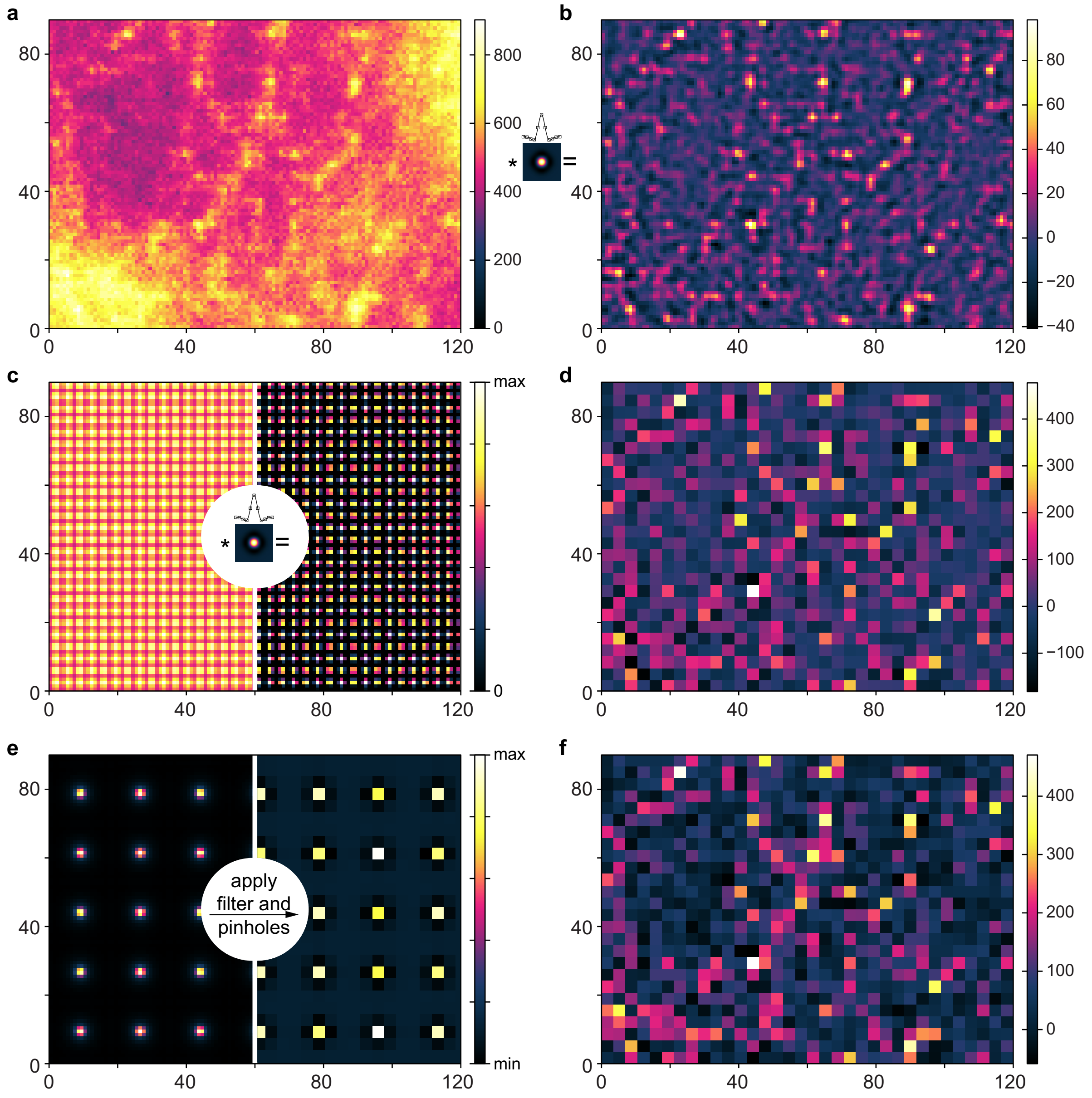

Supplementary Figure 3 | Spatial bandpass filtering and the extraction of the signals emitted at the positions of the nulls. (**a**) An acquired camera image in a small region of 120×90 pixels (about 35×26 illumination periods). The analog-to-digital converter (ADC) bias was subtracted and the ADC conversion factor was applied to scale the image in photo-electrons. (**b**) Bandpass filtered image obtained by convolving the acquired image **a** with the Laplacian-of-Gaussian filter kernel shown to scale in-between the images. (**c**) Determination of the pinholes and sensitivities. Left: Simulated image of equally bright emitters at every position of a null. Right: Bandpass filtered simulated image with applied zero threshold. Positive values are identifying the pinhole regions (4–6 pixels within a 3×3 neighbourhood at the nulls). (**d**) Extracted signals at the nulls obtained by the integrated signal in each pinhole normalized with its sensitivity. (**e**) The pinhole PSFs are extracted from simulated and analysed images. Equally bright emitters are placed at every fifth null to build all PSFs with only 5×5 images. (**f**) The PSF matrix is then applied in a least squares conjugate gradient fit to find the closest matching pinhole signals (shown) that yield the observed image **d**.

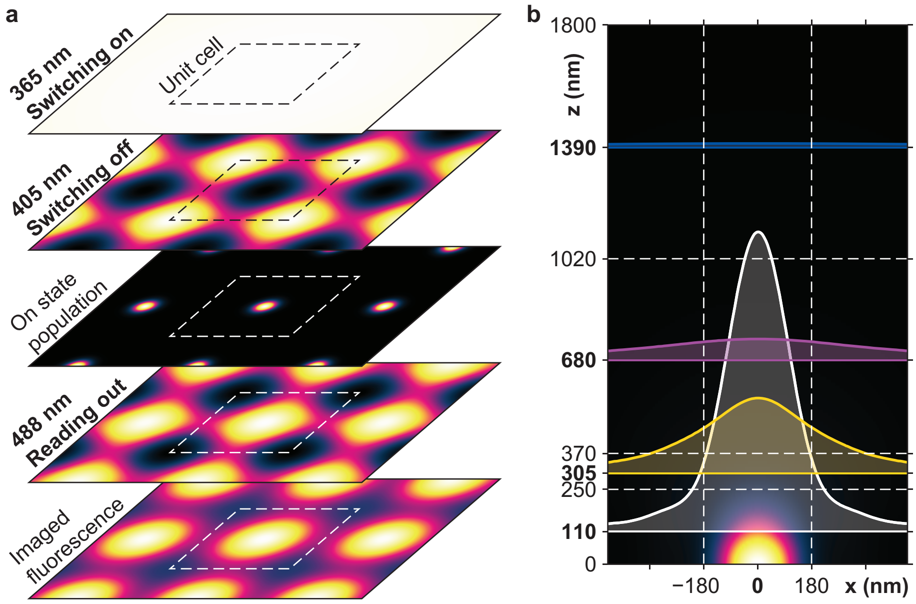

Supplementary Figure 4 | Imaging PSF for the model-based RESOLFT image reconstruction. (**a**) Estimation of the fluorescence image based on the illumination patterns and illumination doses when switching and exciting Dreiklang. The on state population and the read-out energy determine the potential fluorescence emission. The lowest panel illustrates the fluorescence image in the focal plane. (**b**) Estimation of the imaging PSF and separation into a small number of axial sections. The focal section extended from the cover-slip interface ($z = 0$ nm) into the sample such that its PSF area was $\sqrt{2}\times$ the focal PSF area at cover-slip interface. Adjacent sections were placed such that their PSF areas quadrupled from section to section. Dashed lines indicate the section borders and the lateral extent of the unit cell. The mutually distinct section PSFs are illustrated by cross-sections.

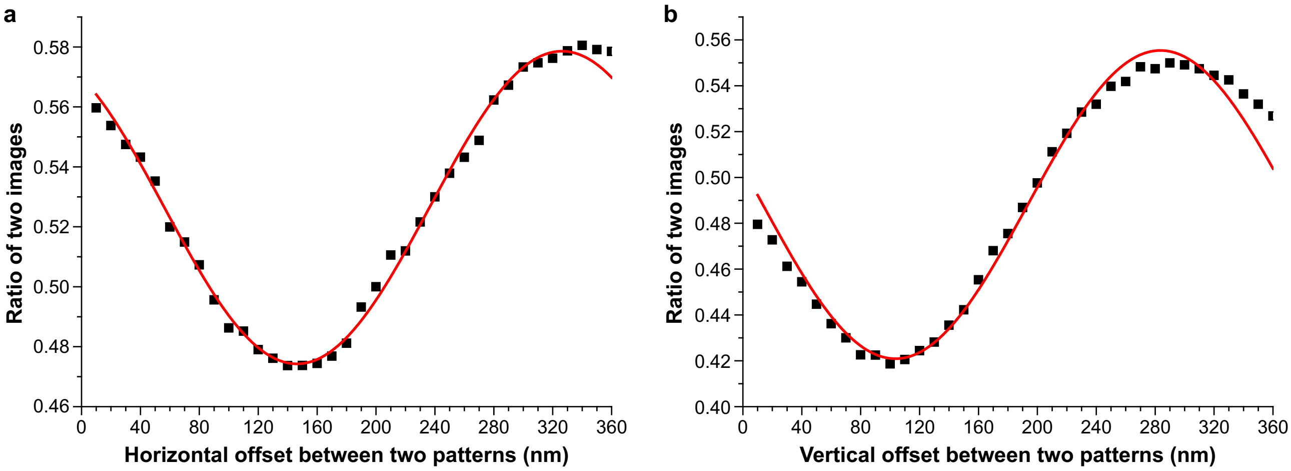

Supplementary Figure 5 | Determination of offset between patterns due to phase shift. The offsets between patterns of 405 and 488 nm light for horizontal (**a**) and vertical (**b**) components are determined separately by blocking the other component (no light in the orthogonal pattern). A sequence of two images each, of dense keratin19–Dreiklang structures, were taken, with activation, off-switching and read-out illuminations. For the first image, the pattern was displaced by half a period (180 nm) between the off-switching and the read-out step, as would be suitable if these patterns overlapped completely. For the second image, an additional variable shift of 10–360 nm in steps of 10 nm was introduced. Fluorescence in the recorded images was therefore modulated due to variable spatial coincidence of switch-off and read-out, with it being minimal when the two patterns (off-switching and read-out) were completely overlapping, and being maximal when completely out-of-phase. From this ratio we determined the relative offset between the patterns of two wavelengths and took it into account for later measurements. Black dots represent the ratios of the mean fluorescence in the images with the offset to the mean fluorescence in the images without the offset. Red lines represent the fit to sinusoidal functions with 360 nm period. The maximal ratio was found at the optimal offsets of 320 nm (**a**) and 280 nm (**b**).

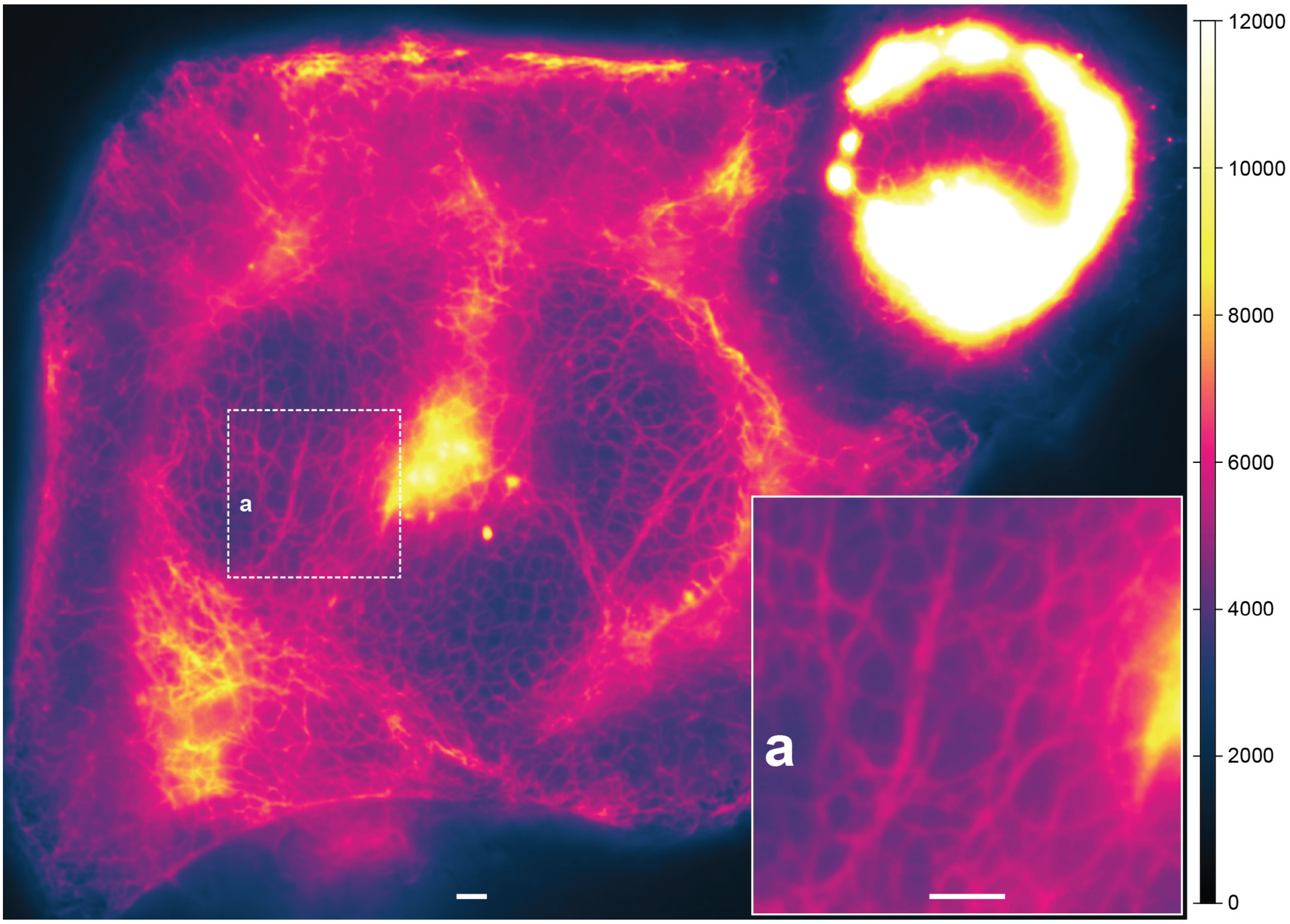

Supplementary Figure 6 | Wide-field image obtained by shifting and summing the acquired images. The image brightness was saturated at a maximum of 12’000 for clarity (peak brightness 121’000). (**a**) 2.5× magnified region. Scale bars: 2 µm. Displayed fields of view: 78×61 µm^2, 11×11 µm^2 (**a**).

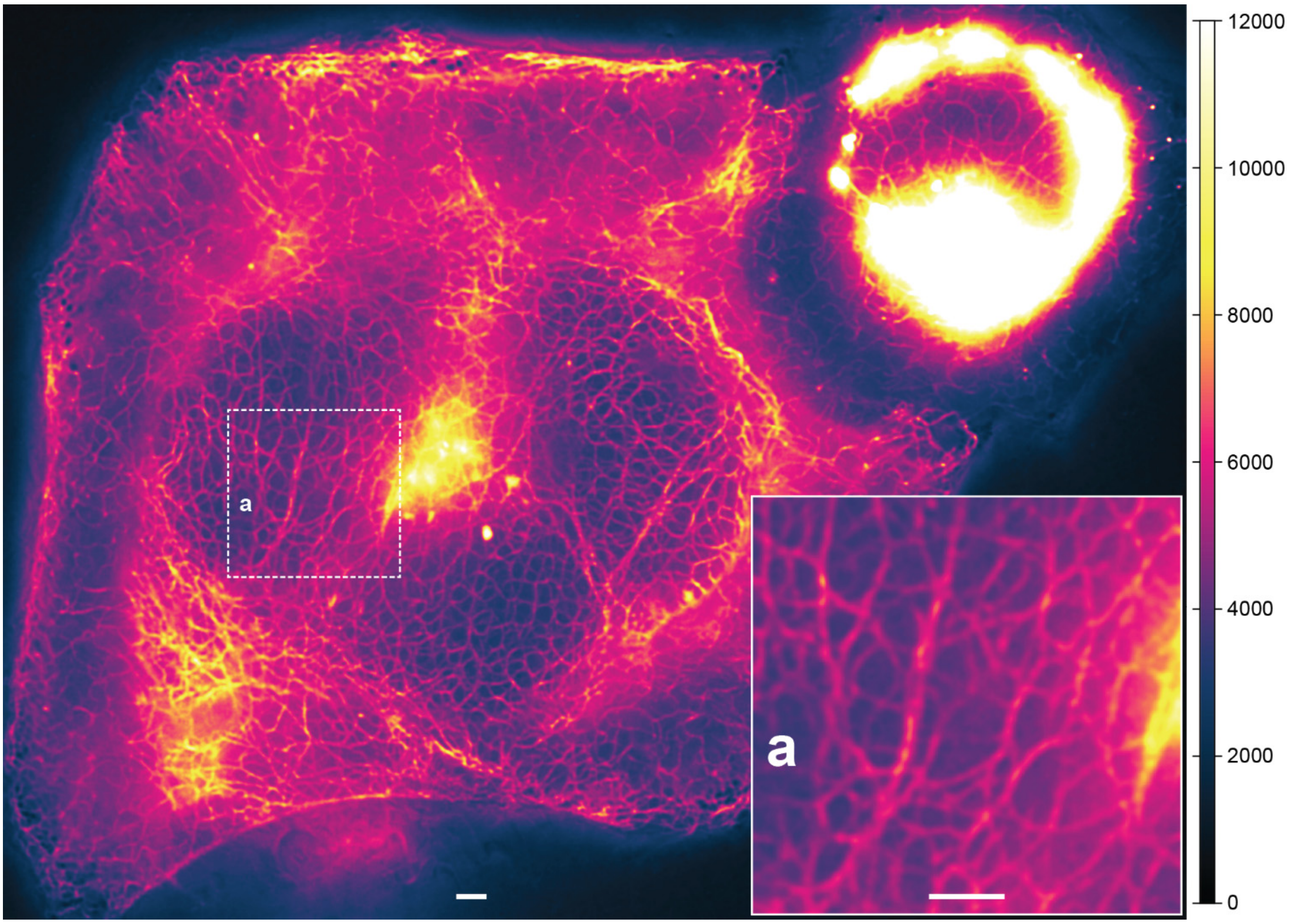

Supplementary Figure 7 | Deconvolved wide-field image as shown in Supplementary Figure 6. The image brightness was saturated at a maximum of 12’000 for clarity (peak brightness 153’000). (**a**) 2.5× magnified region. Scale bars: 2 µm. Displayed fields of view: 78×61 µm^2, 11×11 µm^2 (**a**).

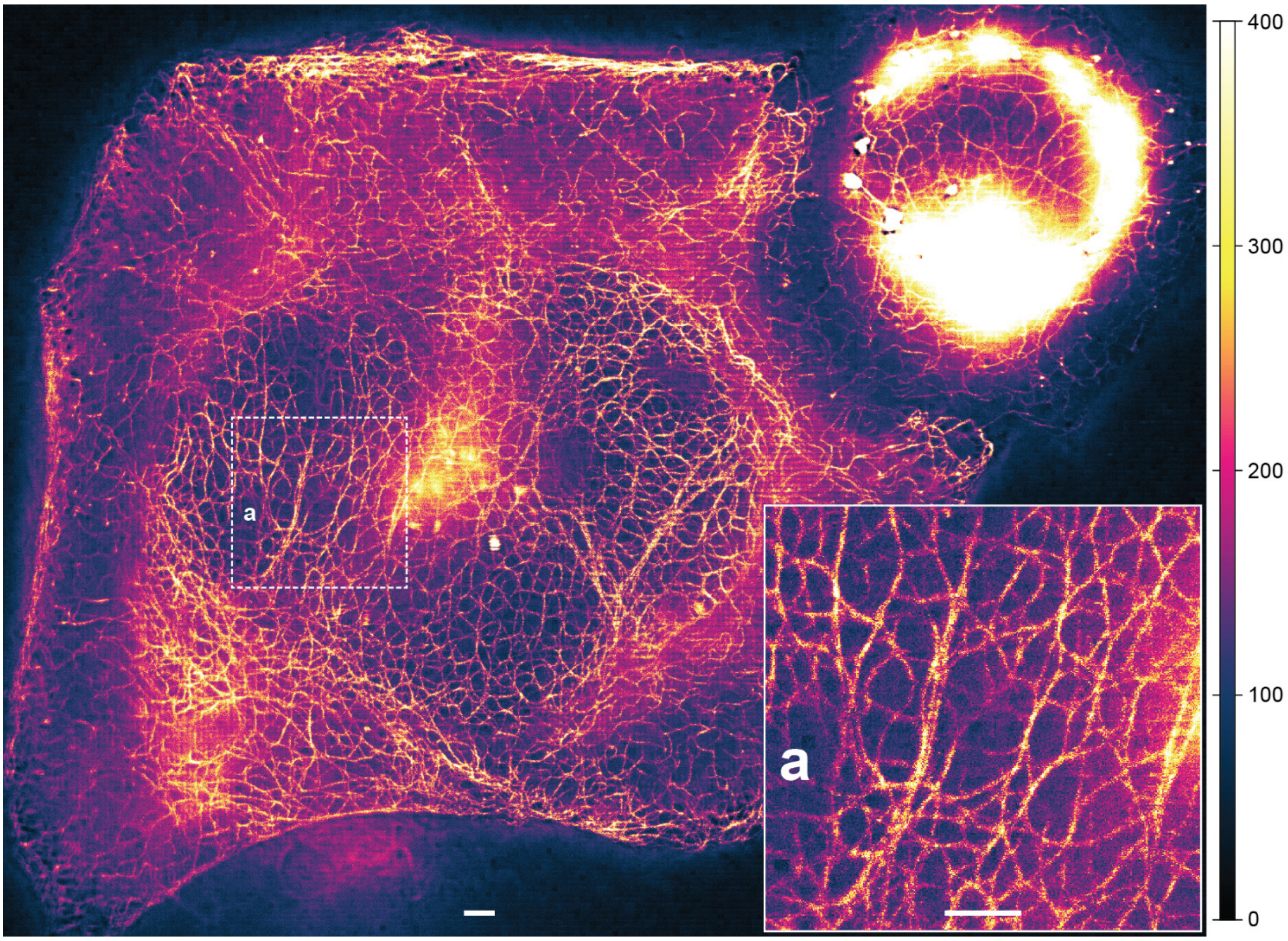

Supplementary Figure 8 | Parallelized RESOLFT analysis with local pinholes. The image brightness was saturated at a maximum of 400 and negative values were ignored for clarity (peak brightness 8'090). (**a**) 2.5× magnified region. Scale bars: 2 µm. Displayed fields of view: 78×61 μm^2, 11×11 μm^2 (**a**).

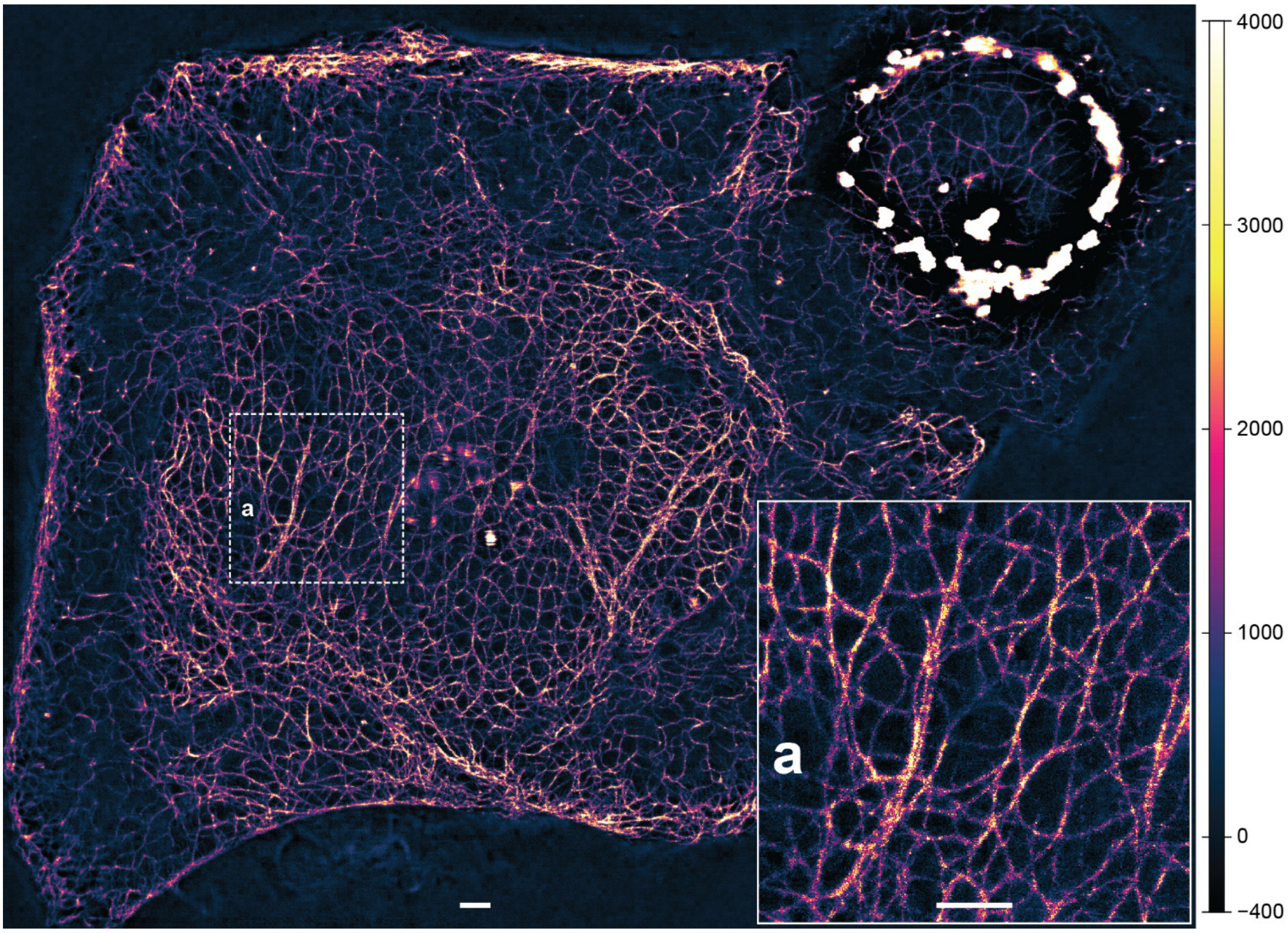

Supplementary Figure 9 | Background elimination by spatial bandpass filtering of the acquired images. The RESOLFT image brightness was saturated at a maximum of 4’000 for clarity (peak brightness 130’000). (**a**) 2.5× magnified region. Scale bars: 2 µm. Displayed fields of view: 78×61 µm^2, 11×11 µm^2 (**a**).

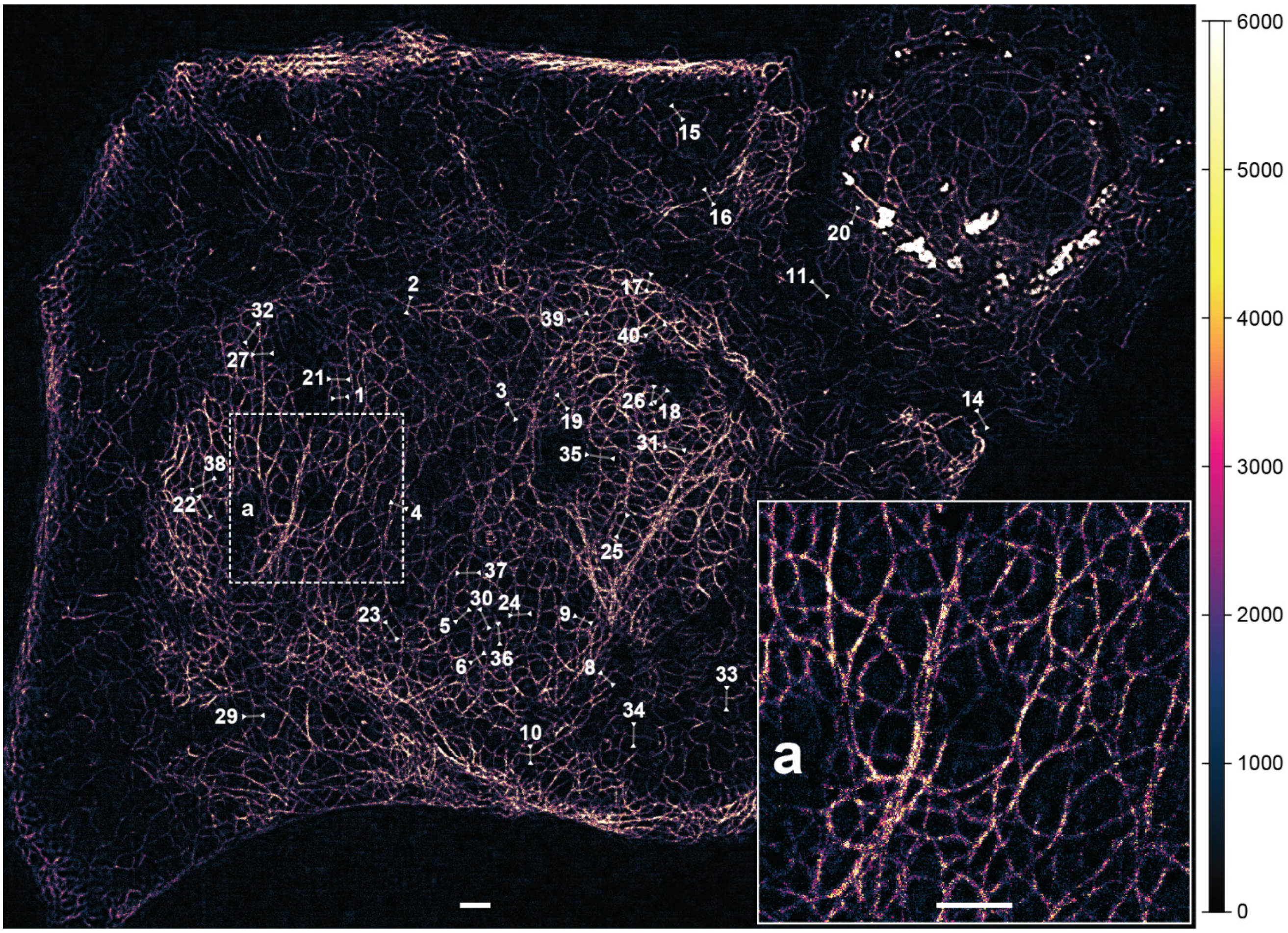

Supplementary Figure 10 | In-focus image obtained by the model-based RESOLFT image reconstruction. The image brightness was saturated at a maximum of 6’000 for clarity (peak brightness 164’000). Line profiles are indicated for **Supplementary Figure 11**. (**a**) 2.5× magnified region. Scale bars: 2 µm.

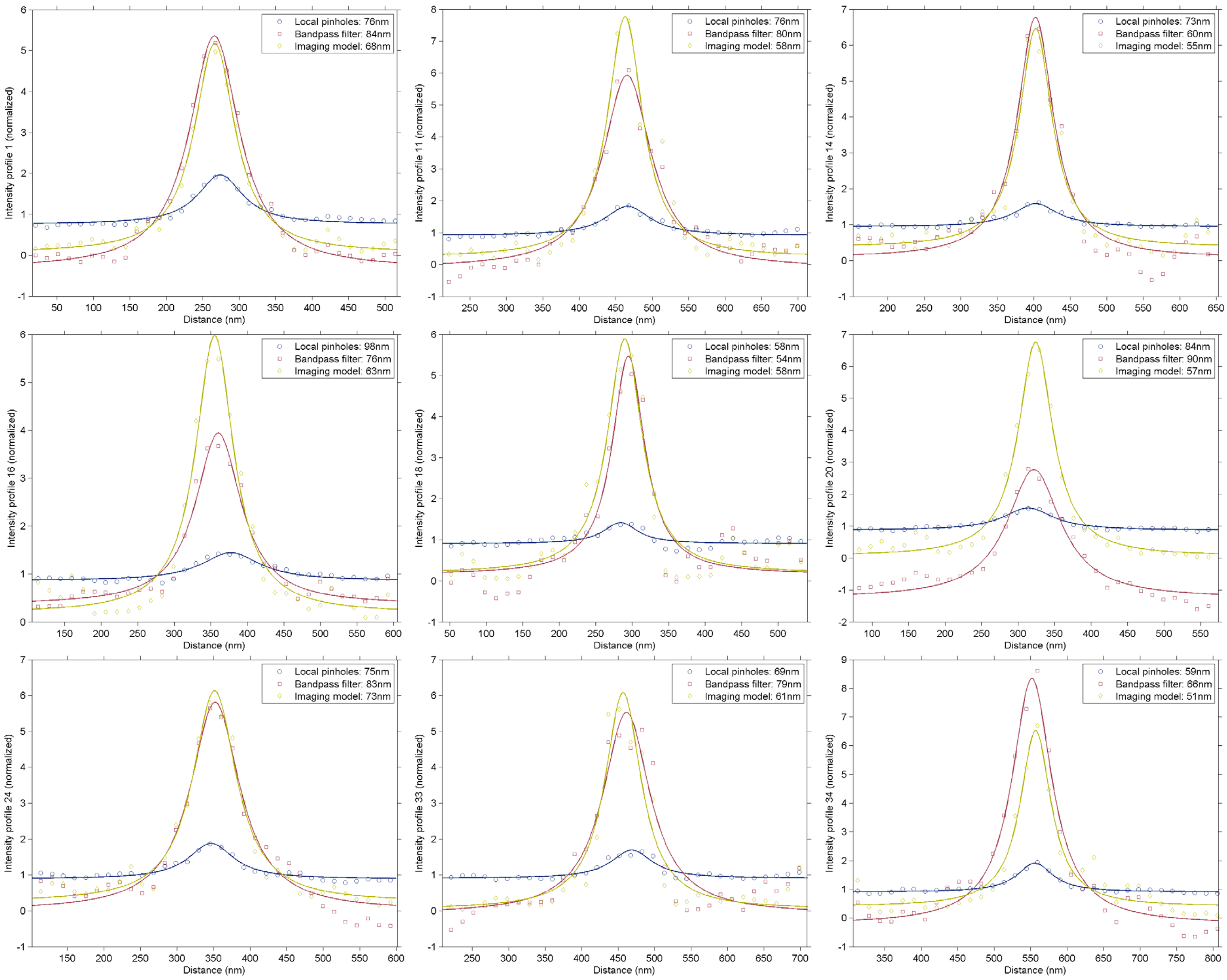

Supplementary Figure 11 | Line profiles across keratin19 filaments in Supplementary Figure 8–10. The intensity line profiles (dots) were averaged over a width of 5 pixels (130 nm) for the different RESOLFT analysis methods and then normalized by the averages of their absolute values. The positions of the line profiles are indicated in **Supplementary Figure 10**. Fits to a Lorentz function are shown as solid lines and the corresponding FWHM values are reported. By looking for clearly visible but likely single tubulin filaments, we selected 20 profiles in the local pinholes RESOLFT reconstruction and another 20 profiles in the imaging model RESOLFT reconstruction. From these 40 profiles we excluded four near-duplicates. The model-based analysis performed best with nine profiles (25%) measured at 51–62 nm FWHM and a median FWHM of 76 nm. For the bandpass filtered analysis, nine profiles were measured at 54–80 nm FWHM, the median was 94 nm. The corresponding values for the local pinholes analysis were 58–84 nm FWHM and 104 nm.

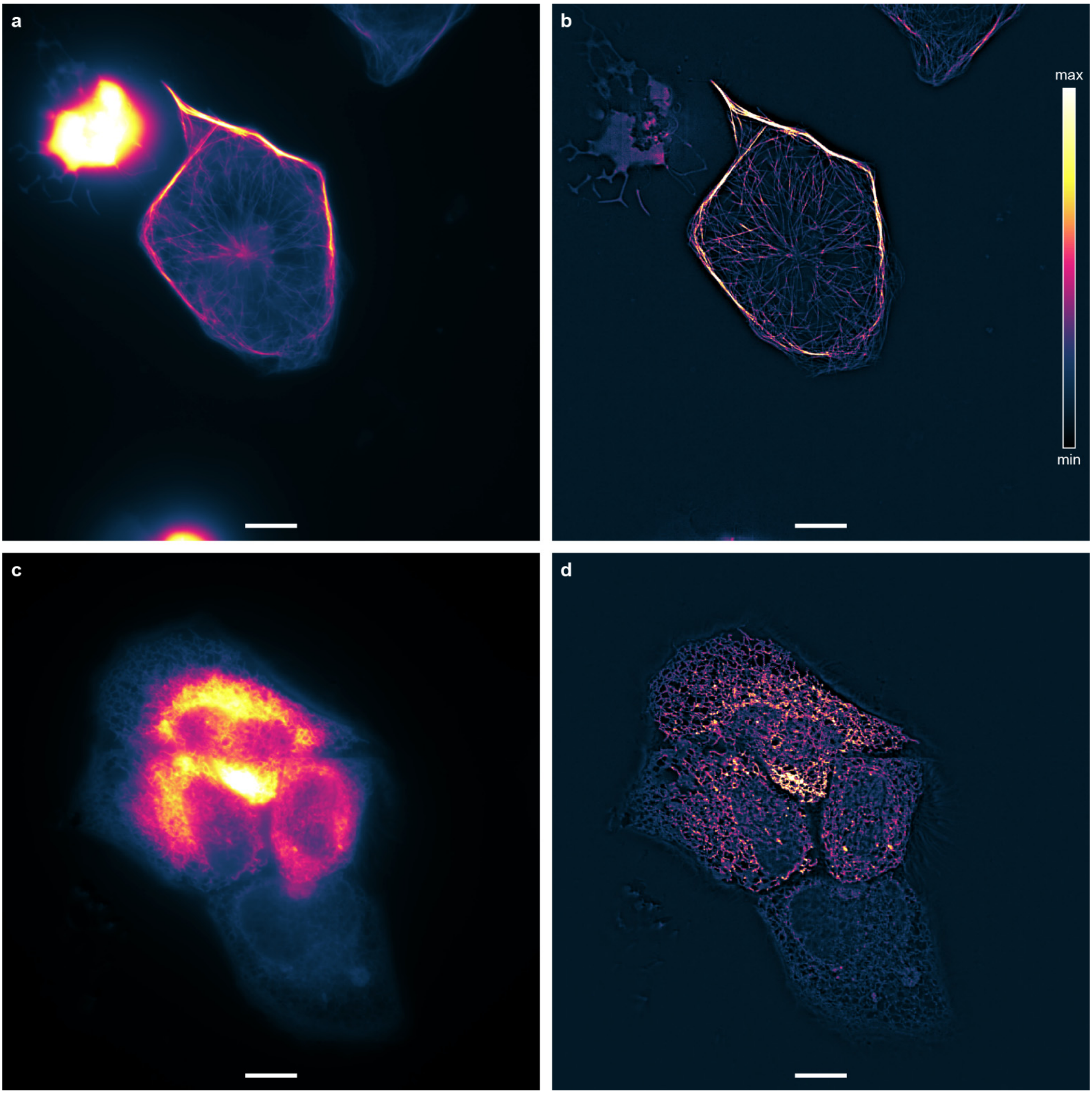

Supplementary Figure 12 | Full images for magnified regions in Fig. 2k–n. Wide-field images (**a**,**c**) and RESOLFT reconstruction with bandpass filtering (**b**,**d**) of HeLa cells expressing MAP2–Dreiklang (**a**,**b**), imaged with 12×12 scan steps of 30 nm step length, and KDEL–Dreiklang (**c**,**d**), imaged with 8×8 scan steps of 45 nm. Displayed is the acquired field of view: 104×104 µm^2. Scale bars: 10 µm.

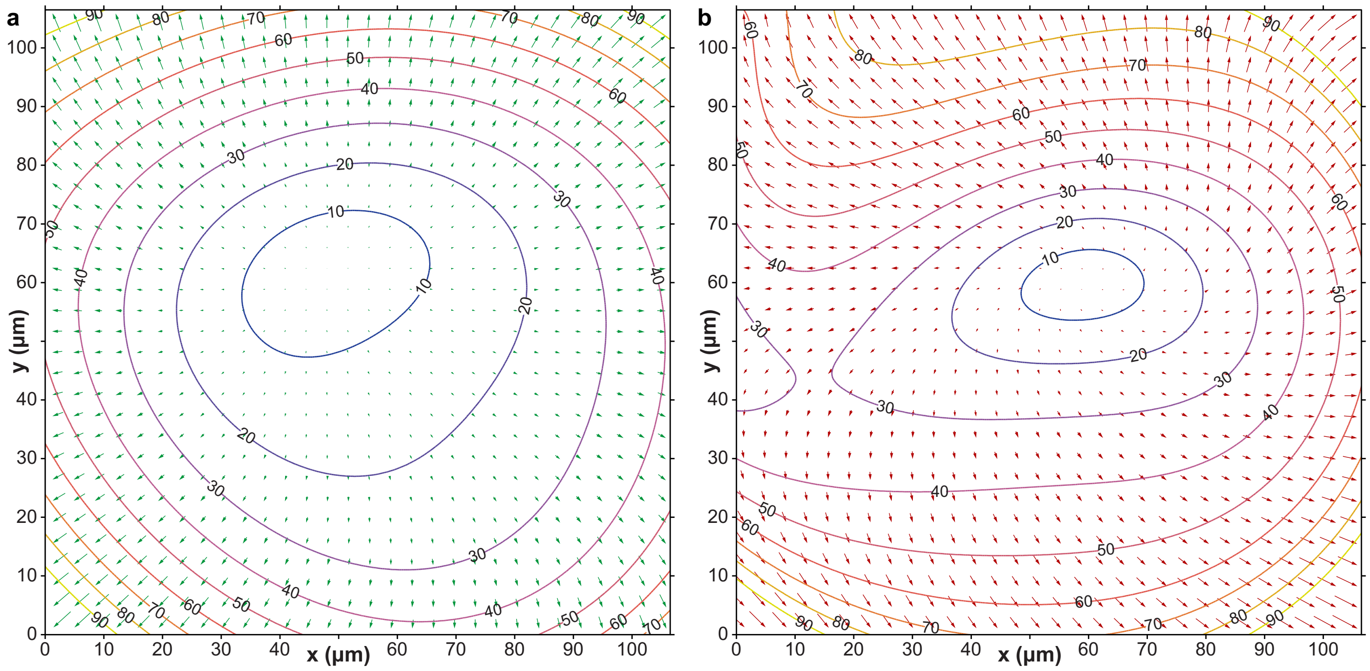

Supplementary Figure 13 | Mismatches of illumination patterns estimated by images of fluorescent layers. The contour lines show the relative shift of the fluorescence peak positions of the 488 nm (**a**) and 592 nm (**b**) illuminations versus the 405 nm illumination in percent of the average horizontal and vertical pattern period. The arrows point at every 10th peak of the 405 nm illumination pattern and indicate the distance from the corresponding illumination peak of the other patterns. The lengths of the arrows are magnified 10× to match the increased peak spacing. We deem acceptable horizontal and vertical mismatches of up to 30–40% and, respectively, up to 45–55% along the diagonal directions.

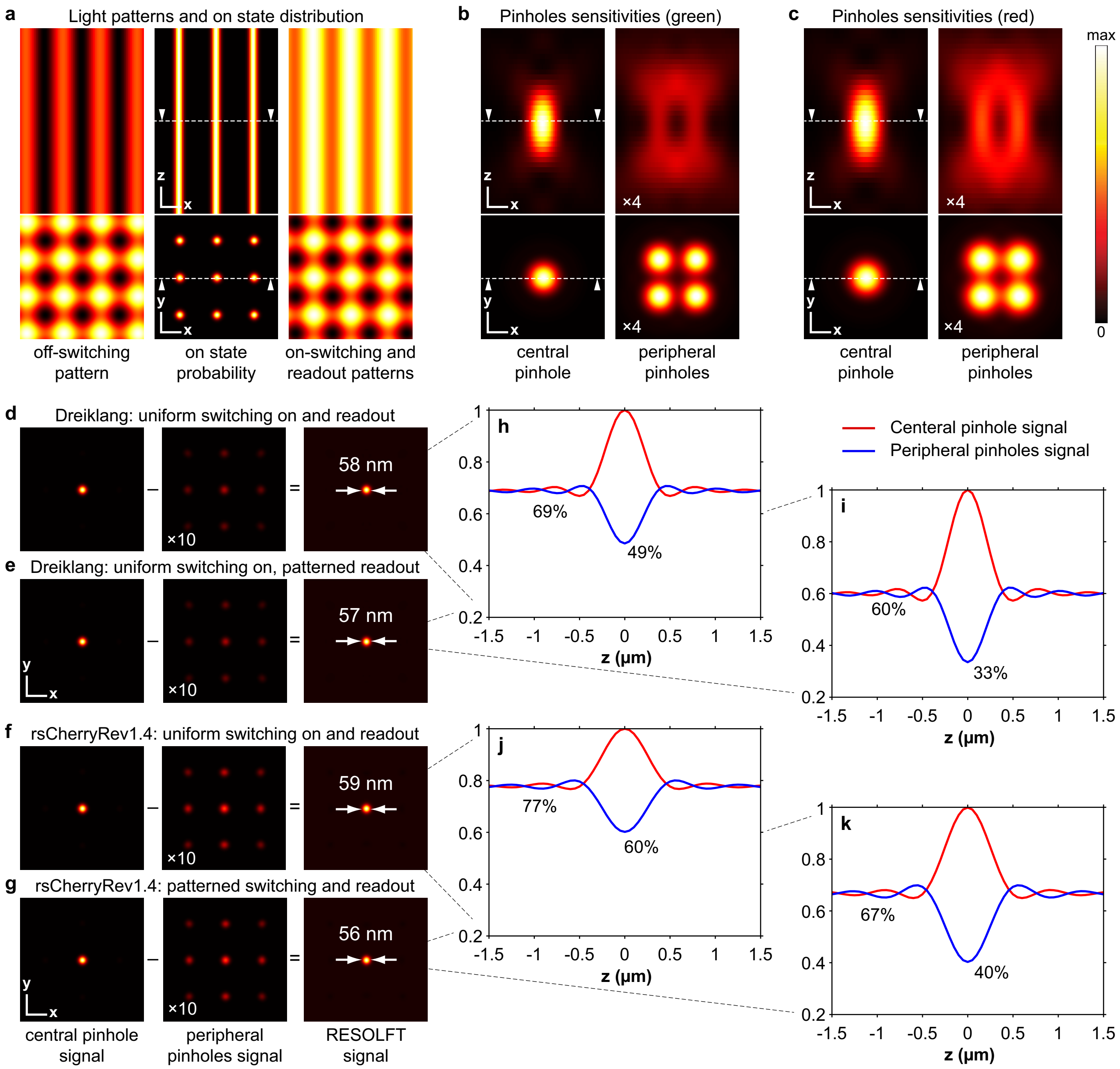

Supplementary Figure 14 | Calculated lateral resolution and background rejection with and without patterned activation and readout. (**a**) Cross-sections of the off-switching illumination pattern (Λ = 360 nm) (left); potential population of fluorophores in the on state after switching off (center); and cross-sections of the on-switching and readout illumination patterns, shifted half a period with respect to the off-switching pattern (right). (**b**,**c**) Cross-sections of the fluorescence detection PSF by a small pinhole at a null (left) or four pinholes around the null (right) for a fluorescence wavelength of 520 nm for Dreiklang **b** and 610 nm for rsCherryRev1.4 **c**. (**d**–**g**) Effective imaging PSF (right) as obtained by measuring the central pinhole signal (left) and subtracting the peripheral pinholes signals (center) for fluorophores in the focal plane. Effective PSFs upon imaging Dreiklang and rsCherryRev1.4 with and without patterned on-switching and readout are illustrated and their lateral FWHMs are indicated. (**h**–**k**) Corresponding normalized axial responses of the central and peripheral pinholes for fluorophores located in a thin layer at different axial distances from the focal plane. The background levels and the minimum peripheral signals at the focal plane are indicated. The signals of the peripheral pinholes are scaled up 4× (**b**,**c**) or 10× (**d**–**g**) for clarity. Scale bars (axes indicators): 200 nm.

Supplementary Methods

Optical setup

We adapted the wide-field RESOLFT instrument by Abberior Instruments, whose original optical setup is largely based on setup described in [1]. The modified RESOLFT microscope is shown in **Supplementary Figure 1**. An Olympus IX-83 microscope body was used and customized at its side port. Three continuous-wave laser beams of 405 nm, 488 nm and 592 nm wavelengths were combined by clean-up filters and dichroic mirrors and coupled into a polarization-maintaining single-mode fiber. An achromatic lens L_1 collimated the beams at the fiber output to a beam diameter of about 12 mm. An achromatic half-wave retarder plate (λ/2) and polarizing beam splitters (PBS) sent the beams at equal powers through two binary phase gratings (G_1 and G_2). The phase gratings diffracted the s-polarized beams primarily into the first diffraction orders. These were focused by the achromatic lens L_2 and selected by an order selection mask (OS) to cancel stray light and the order zero. The achromatic lenses L_3 and L_4 relayed the illumination beams on a tip-tilt piezo scan mirror. The custom-made dichroic mirror D_1 transmitted the wavelength ranges of 350–430 nm, 480–490 nm and 588–598 nm. The achromatic lens L_5 and the microscope tube lens relayed the beams into the entrance pupil of the microscope objective. Thereby, 50× demagnified images of the phase gratings were produced at the objective's focal plane. Hence, the sample was illuminated with square grids of 360 nm periods. When imaging Dreiklang, we switched the protein's fluorescence on with a homogeneous illumination of 365 nm wavelength provided by a LED source and injected via the dichroic mirror D_3. The fluorescence was collected by the microscope objective and sent back via the scan mirror to the main dichroic mirror D_1. The lenses L_5 and L_4 and the achromatic lenses L_6 and L_7/L_8 relayed the intermediate image onto the sensors of two scientific CMOS cameras. The wavelength ranges of 440–470 nm, 500–575 nm and 610–750 nm were reflected by D_1 and further split by the dichroic mirror D_2 into two colour channels. The dichroic mirrors and the bandpass filters F_1 and F_2 selected the fluorescence at 500–575 nm and 610–680 nm wavelengths.

RESOLFT image reconstruction

The raw wide-field images were analysed in three major steps:

1. Determination of the signal origins corresponding to the positions of the intensity minima ("nulls") of the switching off illumination pattern.
2. Estimation of the in-focus fluorescence emission from the regions of the nulls.
3. Reattribution of the fluorescence emission to confined regions at the nulls.

We improved the image reconstruction over the previously published method [1] to (i) more accurately identify the positions of the nulls by accommodating distortions of the illumination pattern and/or its images, and to (ii) better reject the fluorescence emission from out-of-focus features. The analysis can exclude sensor pixels with high dark current or excessive read noise as identified in dark pictures. The new analysis algorithms are detailed below.

1. Signal origins

Whenever possible, the set of positions $\boldsymbol{X} = \{(x_{mn}, y_{mn})\}$ of the nulls were extracted from the raw images of a measurement (**Supplementary Figure 2**). For samples with very sparse features, an extra calibration measurement of a thin layer of fluorescent proteins was performed shortly prior to the image acquisition of the sample.

We assume that the nulls are located on an approximately regular square grid aligned approximately with the camera sensor along its horizontal x and vertical y directions. The nulls are projected at the same sensor positions for the image frames captured during a measurement. Therefore, the raw images of an entire acquisition were summed to obtain $I(x, y)$, an image of the average fluorescence emission in all unit cells across the field of view (**Supplementary Figure 2a**).

The grid periods Λ_x and Λ_y were estimated by identifying the spatial frequency of the major peak in the horizontal and vertical spatial power spectra of the sum image (**Supplementary Figure 2b**). These spectra were calculated with the Fourier transform with a frequency sampling fine enough to achieve a precision of about three digits in the period estimates (we used 128× oversampling). To account for potential misalignments and distortions of the illumination grid, the power spectra were calculated on moving averages spanning 30 pixels along the opposite direction (**Supplementary Figure 2a**). The periods were then estimated by a weighted average on the peak spatial frequencies, where the weights were proportional to the peak spectral power density (**Supplementary Figure 2b**).

Next, local offsets of the signal origins were determined by correlating the sum image $I(x, y)$ with the estimated x and y grid in neighbourhoods of 20×20 periods extent (**Supplementary Figure 2c**). For the horizontal position offsets, the sum image was multiplied by $F_x(x) = \exp(2i\pi x/\Lambda_x)$ and correlated with a pyramidal mask $W(x, y) = \max(0; 1 - |x/10\Lambda_x|) \times \max(0; 1 - |y/10\Lambda_y|)$:

$$C_x(x, y) = (I(x, y)F_x(x)) * W(x, y) \quad (1)$$

Pixel by pixel, the phase $\phi_x(x, y) = \arg C_x(x, y)$ of this moving correlation (**Supplementary Figure 2d**) indicates the position offset $\Delta x(x, y) = \phi_x(x, y)\Lambda_x/2\pi$ of the local null with respect to the grid $F_x(x)$. In order to locate the signal origins unambiguously and reduce the phase noise, the phase $\phi_x(x, y)$ was unwrapped (c.f. algorithm by Herráez et al. [2]) and the resulting phase map $\Phi_x(x, y)$ was low-pass filtered by the first 25 fringe Zernike polynomials with weights $|C_x(x, y)|$ (**Supplementary Figure 2e**). The Zernike polynomials allowed to extrapolate the phase map in the border region of partial overlap of $I(x, y)F_x(x)$ and $W(x, y)$. The vertical position offsets were found analogously using the grid pattern $F_y(y) = \exp(2i\pi y/\Lambda_y)$ along the vertical direction to estimate $\Delta y(x, y)$.

The signal origins $\boldsymbol{X}$ (**Supplementary Figure 2f**) were then obtained by shifting the initial positions $(x_{mn,0}, y_{mn,0}) = (m\Lambda_x, n\Lambda_y)$, with integer m and n, by the position offsets $(\Delta x, \Delta y)(x_{mn,0}, y_{mn,0})$. To reduce the error in case of significant offsets, the position estimates were updated several times with only a fraction of the total offset:

$$(x_{nm,j}, y_{mn,j}) = (x_{mn,j-1}, y_{mn,j-1}) + \frac{(\Delta x, \Delta y)(x_{mn,j-1}, y_{mn,j-1})}{J} \quad \forall j \in \{1,2, \dots, J\} \quad (2)$$

Within regions of sample features, this local refinement of the position estimates allowed to accommodate for errors of up to a few percent in the estimation of the grid periods. Moreover, it accounted for small misalignments of the illumination patterns with the camera's sensor axes. Simulations showed that the signal origins were reliably found with less than one tenth of a period position error for sum images with SNR > 2.

2. Signal retrieval

Signal and background estimation by local pinholes

Signals were locally integrated using Gaussian-weighted masks to include neighbouring pixels at the nulls. For each scan step, the local background at each null was determined by 80% of the mean of the signals obtained at the four nearest-neighbour positions diagonally in between the nulls, and then subtracted. For further details of the computational image reconstruction see [1].

Background elimination by spatial bandpass filtering

Supplementary Figure 3 illustrates the image analysis. Each raw image (**Supplementary Figure 3a**) was smoothed with a Gaussian filter of FWHM $= (\Lambda_x + \Lambda_y)/(4\sqrt{2})$ for reducing noise and the Laplacian was taken from the smoothed images to eliminate the background (**Supplementary Figure 3b**). These bandpass-filtered raw images were then integrated in small regions (pinholes) at the nulls' positions $\boldsymbol{X}$ to estimate the fluorescence emitted there (**Supplementary Figure 3d**). The pinholes were determined by the positive signals in 3×3 camera pixels neighbourhoods centred at the nulls (**Supplementary Figure 3c**). In order to equalize their sensitivities, the pinholes' signals were determined by processing a simulated image of equally bright emitters at the nulls.

This Laplacian-of-Gaussian filtering eliminated background efficiently but introduced some artefacts due to partially overlapping images of adjacent nulls (observe for instance negative values in **Supplementary Figure 3b,d**). Therefore, images of fluorescence emitters in the focal section at single nulls were simulated (described in the next section) and processed to estimate the point spread functions including the image analysis (**Supplementary Figure 3**e). The artefacts were then reduced by redistributing the pinhole signals (**Supplementary Figure 3c**) such that the squared difference to the observed bandpass filtered image (**Supplementary Figure 3b**) was minimized (**Supplementary Figure 3f**).

Model-based maximum likelihood estimation of signals

Given the parameters of the microscope objective and the illumination optics, the illumination patterns in a lateral plane of the sample were estimated with the fast focus field calculation tool [3] (**Supplementary Figure 4**).

The on- and off-switching and the emission of the fluorescent protein is illustrated in **Supplementary Figure 4a** and was estimated as described in the RESOLFT section in reference [4]. We assumed to switch on up to 80% of the proteins ($n_{\text{on}} < 1.6$).[1] We assumed an off-switching illumination dose to reach a five-fold improvement in lateral resolution ($n_{\text{off}} < 25$), which corresponded to 60–70 nm FWHM of the regions with emissive fluorescent proteins. We further assumed to switch off up to 80% during read-out of the fluorescence ($n_{\text{det}} < 1.6$) for rsCherryRev1.4 and up to 25% ($n_{\text{det}} < 0.3$) for Dreiklang.[2]

The detection point spread function (PSF) was estimated with the calculation described by Leutenegger and Lasser [5]. The detection PSF was first calculated in lateral planes with a resolution of 2 nm over an axial range of 2 µm in steps of 10 nm. Neighbouring lateral planes were then grouped into sections such that the areas of the PSFs in adjacent sections quadrupled (**Supplementary Figure 4b**). The planes of the focal section were grouped such that the focal PSF area increased by 42% with respect to the smallest PSF area in the focal plane. Thus, a set of significantly different PSF sections $\text{PSF}_i(x, y)$ was obtained, centered at axial positions z_i and spanning an axial range Δz_i. In particular, we used $z \in \{110,305,680,1390\}$ nm and $\Delta z \in \{250,120,650,780\}$ nm for modelling the focal section at the glass cover slip and three neighbouring sections in the sample. The fluorescence signal from a unit cell – calculated as outlined in the previous paragraph – was then convoluted with the section PSFs to estimate the images of features in these sections.

The image formation model $\vec{y} = \boldsymbol{M}\vec{c} + \vec{\varepsilon}$ consisted of a sparse matrix $\boldsymbol{M} = (M_{mn})$, whose element M_{mn} was the contribution to pixel m by the PSF of the nth coefficient. The elements (c_n) of the coefficient column vector $\vec{c}$ were the estimated fluorescence signals at the positions $\boldsymbol{X}$ in the sections i. The measured image in column vector $\vec{y}$ was thus obtained by the matrix product $\boldsymbol{M}\vec{c}$ plus shot noise and read-out noise $\vec{\varepsilon}$. In order to build the model $\boldsymbol{M}$ column by column, the section PSFs were placed at the lateral positions $\boldsymbol{X}$ and binned into the camera pixels. Thereby, sub-pixel shifts of the nulls' positions with respect to the camera pixels were taken into account.

A conjugate gradient solver was used to find the coefficients $\vec{c}$ maximizing the Poisson likelihood (photo-electron statistics) that the estimated image $\boldsymbol{M}\vec{c}$ corresponds to the measured raw image $\vec{y}$. This fitting procedure eliminated the blurred signals of defocused features. However, only the coefficients of the focal section could be estimated with high spatial resolution as the others showed significant artefacts due to the overlap of the signals of adjacent nulls. The iterative fitting took about an hour per 100 raw images of a RESOLFT image acquisition.

3. Signal reattribution

The retrieved signals were combined into the RESOLFT image by placing each signal at its new pixel position in the final image. Photo-bleaching was accounted for by weighing the signals with the inverse of the average signals in each raw image, whereby a second-order polynomial decay of the average signals was assumed to avoid artefacts due to the sample structure.

Wide-field image reconstruction

In order to compare the RESOLFT images with wide-field images, we combined the raw images as follows. We up-sampled the raw images to match the spatial sampling of the RESOLFT image and shifted each raw image to its position within the unit cell of the illumination patterns. These steps were performed by zero-padding and phase-shifting the spatial spectra of the raw images. The resampled raw images were then summed with weights inversely proportional to the average signals in each image to account for photo-bleaching (see 3. Signal reattribution). This wide-field image was further processed by a Lucy-Richardson deconvolution, where we used the theoretical diffraction-limited detection efficiency as PSF.

Supplementary References

1. Chmyrov A, *et al*. Nanoscopy with more than 100,000 'doughnuts'. *Nature Methods* **10**, 737–740 (2013).

---

[1] Notation as in review [4]: n_{on} designates the number of transitions to the bright on state a fluorescent protein could perform if it would remain in its dark off state during the on-switching illumination pulse. After switching on, the off state is depopulated to a fraction of $\exp(-n_{on})$.

[2] Dreiklang switches off slowly upon illumination at 488 nm wavelength.

2. Herráez MA, Burton DR, Lalor MJ, Gdeisat MA. Fast two-dimensional phase-unwrapping algorithm based on sorting by reliability following a noncontinuous path. *Appl. Opt.* **41**, 7437–7444 (2002).
3. Leutenegger M, Rao R, Leitgeb RA, Lasser T. Fast focus field calculations. *Opt. Express* **14**, 11277–11291 (2006).
4. Vandenberg W, Leutenegger M, Lasser T, Hofkens J, Dedecker P. Diffraction-unlimited imaging: from pretty pictures to hard numbers. *Cell Tissue Res.* **360**, 151–178 (2015).
5. Leutenegger M, Lasser T. Detection efficiency in total internal reflection fluorescence microscopy. *Opt. Express* **16**, 8519–8531 (2008).
